# Wheat VQ Motif-Containing Protein VQ25-A Facilitates Leaf Senescence via the Abscisic Acid Pathway

**DOI:** 10.3390/ijms241813839

**Published:** 2023-09-08

**Authors:** Xiao Meng, Mingyue Lu, Zelin Xia, Huilong Li, Duo Liu, Ke Li, Pengcheng Yin, Geng Wang, Chunjiang Zhou

**Affiliations:** Ministry of Education Key Laboratory of Molecular and Cellular Biology, Hebei Collaboration Innovation Center for Cell Signaling and Environmental Adaptation, Hebei Research Center of the Basic Discipline Cell Biology, Hebei Key Laboratory of Molecular and Cellular Biology, College of Life Sciences, Hebei Normal University, Shijiazhuang 050024, China; mengxiao2012@163.com (X.M.); 15931183235@163.com (M.L.); xiazelino@126.com (Z.X.); 17336339485@163.com (H.L.); liuduo15530891980@163.com (D.L.); like@hebtu.edu.cn (K.L.); yinpengcheng@hebtu.edu.cn (P.Y.)

**Keywords:** *Triticum aestivum* (wheat), leaf senescence, abscisic acid, valine-glutamine (VQ) contained gene family, transcriptional regulation factor

## Abstract

Leaf senescence is an important factor affecting the functional transition from nutrient assimilation to nutrient remobilization in crops. The senescence of wheat leaves is of great significance for its yield and quality. In the leaf senescence process, transcriptional regulation is a committed step in integrating various senescence-related signals. Although the plant-specific transcriptional regulation factor valine-glutamine (VQ) gene family is known to participate in different physiological processes, its role in leaf senescence is poorly understood. We isolated TaVQ25-A and studied its function in leaf senescence regulation. *TaVQ25*-*A* was mainly expressed in the roots and leaves of wheat. The TaVQ25-A-GFP fusion protein was localized in the nuclei and cytoplasm of wheat protoplasts. A delayed senescence phenotype was observed after dark and abscisic acid (ABA) treatment in *TaVQ25*-*A*-silenced wheat plants. Conversely, overexpression of *TaVQ25*-*A* accelerated leaf senescence and led to hypersensitivity in ABA-induced leaf senescence in *Arabidopsis*. A WRKY type transcription factor, TaWRKY133, which is tightly related to the ABA pathway and affects the expression of some ABA-related genes, was found to interact with TaVQ25-A both in vitro and in vivo. Results of this study indicate that TaVQ25-A is a positive regulator of ABA-related leaf senescence and can be used as a candidate gene for wheat molecular breeding.

## 1. Introduction

Leaf senescence is the final stage of plant development and an evolutionarily selected developmental process controlled by highly regulated genetic networks [1]. During leaf senescence, intracellular organelles and macromolecules are broken down and degraded, which mainly contributes to the distribution of nutrients from source to sink [2]. Moreover, photosynthetic efficiency decreases, and chlorophyll, lipids, proteins, nucleic acids, and other macromolecules are degraded [3]. Therefore, senescence is an important adaptive mechanism that plants use to improve their survivability and adaptability in specific ecological niches. Some genes are significantly upregulated and downregulated with the senescence process of plants, usually used as senescence marker genes. *AtSAG12* is the most widely used senescence-associated reference gene for characterizing leaf senescence, and the increase in *AtSAG12* protein during leaf senescence is remarkable [4,5]. The expression levels of SAGs increase with leaf aging, while a photosynthetic gene, CHLOROPHYLL A/B BINDING PROTEIN 1 (*CAB1*), has been shown to be downregulated upon senescence [6].

Senescence begins in an age-dependent manner and is also triggered by environmental signals and various phytohormones. Phytohormones play important roles in regulating leaf senescence [7]. Cytokinins (CKs), auxin, and gibberellin (GA) delay leaf senescence, while ethylene, salicylic acid (SA), jasmonic acid (JA), abscisic acid (ABA), brassinosteroid (BR), and strigolactone (SL) accelerate this process [8,9,10,11,12,13,14]. ABA is a sesquiterpenoid and regulates various physiological processes [15,16]. Endogenous ABA content is an important regulatory factor affecting leaf senescence. MdWRKY40 and MdbZIP44 interact with MdABI5 to promote ABA-mediated leaf senescence by enhancing the transcriptional activity of MdABI5 on MdNYE1 and MdNYC1 in apples [17]. OsWRKY53 accelerates leaf senescence by promoting ABA accumulation in rice [18]. In switchgrass (*Panicum virgatum*), PvCCCH69 acts as a negative regulator for leaf senescence, which suppresses ABA synthesis and signaling pathways [19]. The ABA content and ROSs in the early senescence 3 (*es3*) mutant are accumulated, promoting rice leaf senescence [20]. Although it is well known that hormones can regulate leaf senescence, the link between hormone signaling pathways and the mechanisms regulating leaf senescence remain to be investigated.

Valine-glutamine (VQ) motif-containing proteins are plant-specific and form multigene families with 34, 39, 61, 59, 18, and 74 members in *Arabidopsis thaliana* [21], rice (*Oryza sativa* L.) [22], maize (*Zea mays* L.) [23], tobacco (*Nicotiana tabacum* L.) [24], grape (*Vitis vinifera* L.) [25], and soybean [*Glycine max* (L.) *Merr.*] [26], respectively. The VQ family contains a unique and conserved VQ (FxxxVQxxTG) motif that responds to various environmental signals and plays roles in plant defense, growth, and development [27]. As a family of transcriptional regulators, VQ proteins often work in concert with their interacting partners to fine-tune the complex regulatory network that mediates plant growth and stress responses [28]. The WRKY transcription factor (TF) MINI3 (WRKY10) interacts with VQ14 to regulate endosperm development and seed size in *Arabidopsis* [29]. AtVQ23 (AtSIB1) and AtVQ16 (AtMSK1) are required for the plant’s defense responses [30,31]. VQ proteins also interact with other interacting partners, such as PIF4, ABI5, MAPKs, and RING-type E3 ubiquitin ligase, to coordinate diverse physiological processes [32,33,34]. TaVQ4-D interacts with TaMPK3 and TaMPK6 and plays a role in plant drought stress as the phosphorylated substrates of TaMPK3 and TaMPK6 [35]. TaVQ27 acts as a regulator for salt response and regulation [36]. Although several VQ proteins have been functionally characterized, the biological role of specific VQ proteins under given conditions is unknown. Thus far, few studies have reported the involvement of VQ proteins in leaf senescence, especially in wheat (*Triticum aestivum* L.).

In this study, we isolated the *TaVQ25*-*A* gene and analyzed its expression profiles, subcellular localization, and molecular characteristics. *TaVQ25*-*A* overexpression accelerated leaf senescence and led to hypersensitivity to ABA-induced leaf senescence in *Arabidopsis*. Conversely, a delayed senescence phenotype was observed after dark and ABA treatments in *TaVQ25*-*A*-silenced wheat plants. Finally, we screened the interacting protein TaWRKY133 to further elucidate the function of TaVQ25-A. Hence, our findings revealed the functional role of a new senescence-related transcriptional regulation factor, TaVQ25-A, which is a positive and ABA-related regulator of leaf senescence in wheat. This study lays a solid foundation for elucidating the wheat leaf senescence mechanism and provides a potentially useful regulator for wheat breeding.

## 2. Results

### 2.1. TaVQ25-A Is a Senescence-Associated Gene

Previous studies have identified 113 *TaVQ* genes in the wheat genome [37]. However, the potential molecular mechanisms of senescence-associated *TaVQ* genes have rarely been reported in wheat. Here, we identified a new *TaVQ* gene, TraesCS4A02G290800, abbreviated as *TaVQ25*-*A* from hexaploid wheat ‘Chinese Spring’. The *TaVQ25*-*A* gene fragment contained a 662-bp open reading frame encoding a 113-amino acid protein with a conserved FxxxVQxLTG domain. TaVQ25-A is significantly different from its paralogous proteins TaVQ25-B (TraesCS4B02G023200) and TaVQ25-D (TraesCS4D02G021000) in the 58–66 amino acid (Figure S1).

**Figure 1:**
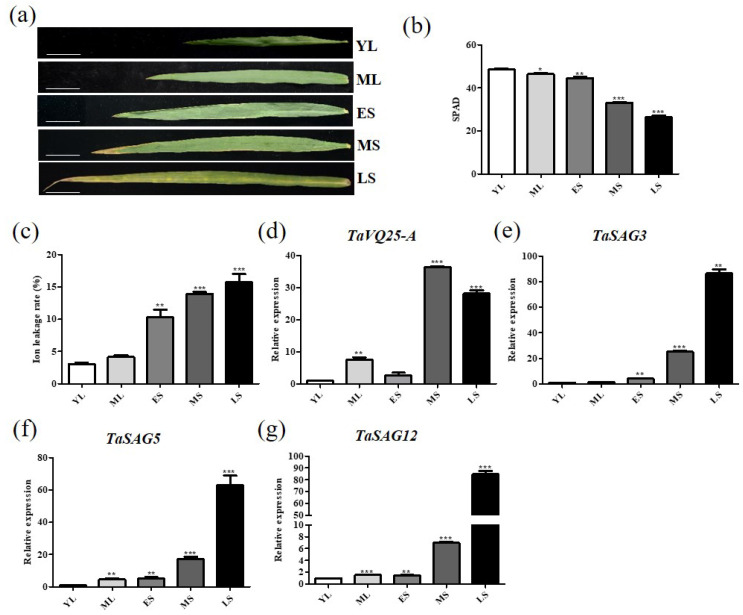
Expression profiles of *TaVQ25*-*A* in wheat flag leaves at different developmental stages. (**a**) Five developmental stages of wheat flag leaves. YL: young leaves; ML: mature and fully expanded leaves; ES: early senescent leaves with <10% leaf area yellowing; MS: middle senescent leaves with leaf area yellowing between ES and LS; LS: late senescent leaves with >50% leaf area yellowing (scale bar: 5 cm). (**b**) Chlorophyll content of leaves in (**a**) was measured by SPAD502 Plus Chlorophyll Meter. (**c**) Ion leakage rate of wheat flag leaves in (**a**). (**d**) Expression level of *TaVQ25-A* at five developmental stages of wheat flag leaves. (**e**,**f**) Expression of the senescence up-regulated marker genes *TaSAG3* (**e**), *TaSAG5* (**f**), and *TaSAG12* (**g**) in wild-type leaves at five developmental stages. Data and error bars represent means ± SE. Student’s *t*-test, * *p* < 0.05, ** *p* < 0.01, *** *p* < 0.001, and *n* ≥ 15. All of the experiments have been repeated at least three times independently.

Based on the high-throughput database WheatOmics (http://202.194.139.32/expression/index.html) (accessed on 15 November 2020), transcripts of *TaVQ25*-*A* were accumulated in senescing leaves. To verify the expression pattern of *TaVQ25*-*A*, we collected wheat flag leaves at five different developmental stages (YL, young leaves; ML, mature and fully expanded leaves; ES, early senescent leaves; MS, middle senescent leaves; LS, late senescent leaves) (Figure 1a). The chlorophyll content and ion leakage rate of these leaves were measured to verify the accuracy of harvesting corresponding leaves (Figure 1b,c). Using qRT-PCR analysis, we proved that the transcripts of TaVQ25-A increased during the progression of leaf senescence (Figure 1d). The expression level of three senescence-upregulated marker genes, TaSAG3, TaSAG5, and TaSAG12, increased gradually in the leaf senescence process (Figure 1e–g). Consistently, the process of leaf senescence proceeds from tip to base, and we measured a higher expression of *TaVQ25*-*A* in the tip than in the base of a single leaf (Figure S2a,b). Our results showed that the lowest chlorophyll content and the highest ion leakage rate were present in the leaf tip (Figure S2c,d). The transcripts of the senescence-upregulated marker gene *TaSAG12* increased gradually from leaf base to tip (Figure S2e). The above data indicate that *TaVQ25*-*A* may be involved in the regulation of wheat leaf senescence.

### 2.2. TaVQ25-A Is Expressed in Different Tissues and Targets the Nuclei and Cytoplasm

To confirm the role of *TaVQ25*-*A* in regulating leaf senescence, we further analyzed the temporal and spatial expression patterns of *TaVQ25*-*A* in a variety of plant tissues, including grain, root, stem, spike, and penultimate, antepenultimate, and flag leaves, using qRT-PCR (Figure S3a). The data showed that the *TaVQ25*-*A* transcripts were most abundant in root and flag leaves (Figure S3b).

**Figure 2:**
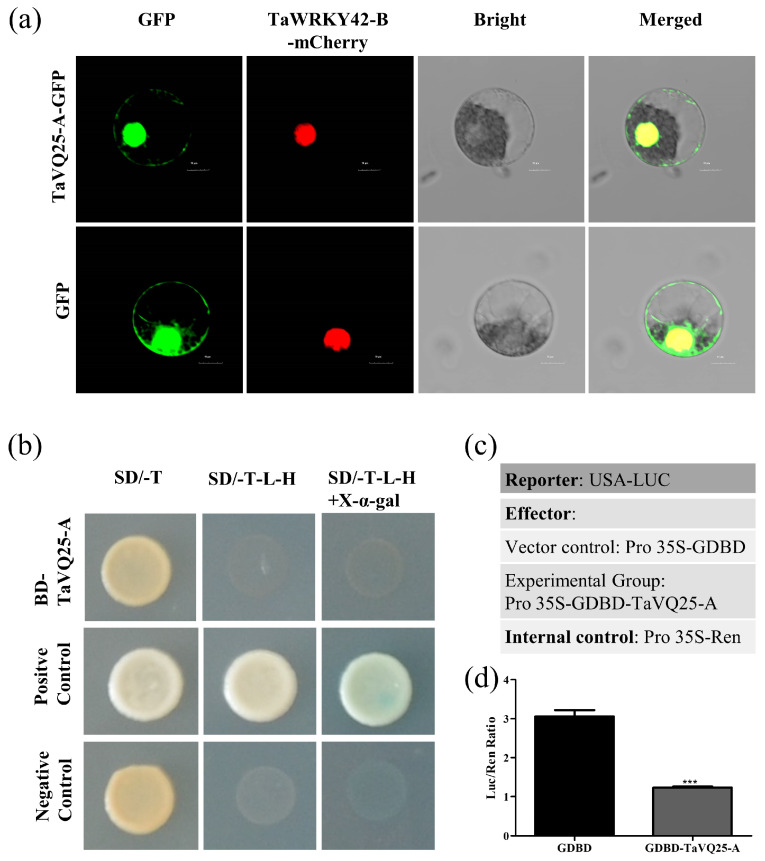
Subcellular localization and transcriptional activation activity of TaVQ25-A. (**a**) Subcellular localization analysis of TaVQ25-A in wheat protoplasts. Column 1 is the signal of GFP; column 2 is the signal of mCherry; column 3 is the bright light field of the same cell; column 4 is the overlaps of GFP, mCherry, and Bright in the same cell. (Scale bar = 10 μm). (**b**) The transcriptional activity of TaVQ25-A was analyzed using the yeast one-hybrid (Y1H) method. (**c**) Schematic diagram of the constructs in the dual luciferase reporter system. (**d**) Measurement of relative Luc reporter activity after transient expression of vectors, as shown in (**c**). Data and error bars represent means ± SE. Student’s *t*-test *** *p* < 0.001, and *n* ≥ 15. All of the experiments have been repeated at least three times independently.

To analyze the subcellular localization of TaVQ25-A, we generated a TaVQ25-A-GFP construct, which was then transformed and transiently expressed into wheat protoplasts. The 35S:GFP vector was used as a control. A TaWRKY42-B-m-Cherry construct was generated and used as a nuclear localization control. Green fluorescence of the GFP signal was observed in the whole wheat protoplast transfected with the empty vector, while the GFP signal of TaVQ25-A-GFP was present in the nuclei and cytoplasm (Figure 2a), which coincided with the red fluorescence of nucleus. This result indicates that the TaVQ25-A protein is localized in the nuclei and cytoplasm.

Furthermore, the GAL4 yeast expression system was used to detect the transcriptional activation activity of TaVQ25-A. The yeast strain AH109 was transformed with the construct pGBTK7-TaVQ25-A, pGBTK7 was used as a negative control, and pGBTK7-TaNAC6 was used as a positive control. TaVQ25-A exhibited no transcriptional activity in yeast (Figure 2b). We also used the luciferase reporter system to explore the transcriptional activity of TaVQ25-A in wheat protoplasts. TaVQ25-A was fused to a Gal4 DNA-binding domain (GDBD) and co-transfected with the firefly luciferase (LUC) gene, and the *Renilla* luciferase (REN) gene, driven by the CaMV 35S promoter fusion was used as an internal control (Figure 2c). The data indicates that TaVQ25-A did not possess transcriptional activity (Figure 2d). These results suggest that TaVQ25-A is localized in the nuclei and cytoplasm and has no transcriptional activation activity.

### 2.3. Overexpression of TaVQ25-A Promotes Age-Triggered Leaf Senescence in Arabidopsis

**Figure 3:**
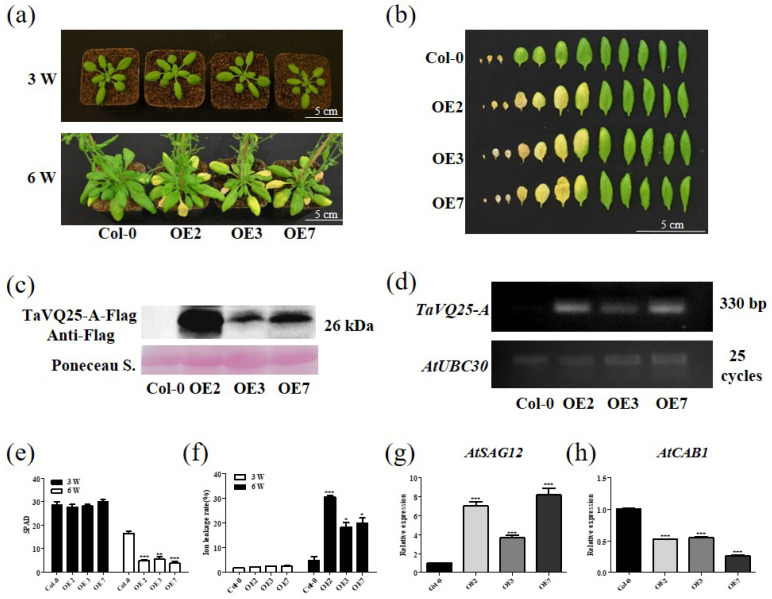
Overexpression of *TaVQ25*-*A* accelerates age-triggered leaf senescence. (**a**) The phenotype of 3-week-old and 6-week-old Col-0 and *TaVQ25*-*A*-OE plants under natural conditions. (**b**) The rosette leaves detached from 6-week-old Col-0 and *TaVQ25*-*A*-OE plants. (**c**,**d**) Protein level (**c**) and transcription level (**d**) of *TaVQ25*-*A* in Col-0 and *TaVQ25*-*A*-OE plants. (**e**,**f**) Chlorophyll content (**e**) and ion leakage rate (**f**) of 3-week-old and 6-week-old Col-0 and *TaVQ25*-*A*-OE plants. (**g**,**h**) The expression of the senescence up-regulated marker gene *AtSAG12* (**g**) and the expression of the senescence down-regulated marker gene *AtCAB1* (**h**) in 6-week-old Col-0 and *TaVQ25*-*A*-OE plants. RNA was extracted from the fifth and sixth leaves of each genotype in (**a**). Transcript levels of the senescence marker genes in Col-0 plants are set to 1. Data and error bars represent means ± SE. Student’s *t*-test, * *p* < 0.05, ** *p* < 0.01, *** *p* < 0.001, and *n* ≥ 20. All of the experiments have been repeated at least three times independently.

To further assess the role of *TaVQ25*-*A* in leaf senescence, we produced *TaVQ25*-*A*-*overexpressing* (OE) lines in *Arabidopsis*. First, we generated a construct in which the fusion of the full-length *TaVQ25*-*A* coding sequence (CDS) and 3 × Flag tags was driven by the CaMV 35S promoter. Three independent homozygous transgenic *Arabidopsis* lines (OE2, OE3, and OE7) were selected for phenotypic and physiological analyses. We observed that 6-week-old OE2, OE3, and OE7 plants showed a precocious leaf senescence phenotype compared to the wild-type (WT) Col-0, but 3-week-old *TaVQ25*-*A*-OE lines showed no obvious difference from Col-0 (Figure 3a). The rosette leaves were harvested from age-matched Col-0 and *TaVQ25*-*A*-OE lines. The leaves of the *TaVQ25*-*A*-OE plants were senesced earlier than Col-0 (Figure 3b). The elevated expression level of *TaVQ25*-*A* was confirmed using RT-PCR and Western blot in *TaVQ25-A*-OE lines (Figure 3c,d). Consistently, the chlorophyll content in the *TaVQ25*-*A*-OE lines was lower than that in Col-0, and the ion leakage rate was accelerated by *TaVQ25*-*A* overexpression (Figure 3e,f). Consistent with the observed phenotypic alterations, the senescence-associated gene (SAG) *AtSAG12* was upregulated in *TaVQ25*-*A*-OE plants, while the senescence-downregulated gene (SDG) *AtCAB1* was suppressed in *TaVQ25*-*A*-OE plants (Figure 3g,h). Therefore, these results suggest that *TaVQ25*-*A* positively regulates the age-triggered leaf senescence process.

### 2.4. TaVQ25-A Promotes Dark-Induced Leaf Senescence

**Figure 4:**
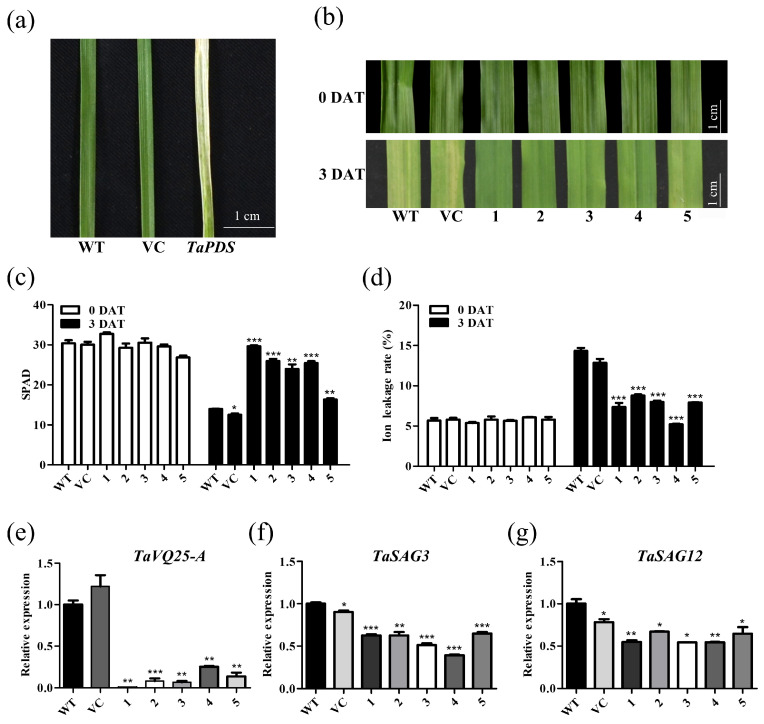
*TaVQ25*-*A*-silenced wheat plants retard the progression of dark-induced leaf senescence. (**a**) Phenotype of virus-induced gene silencing of *TaPDS* in wheat. (**b**) Phenotype changes in the sixth leaf in plants treated with water in the dark. WT: plants infection buffer without BSMV; VC: vector control, plants inoculated with the empty BSMV viral vector; 1–5: plants were inoculated with BSMV targeted to *TaVQ25*-*A*-silenced wheat plants. DAT: days after treatment. (**c**,**d**) Chlorophyll content (**c**) and ion leakage rate (**d**) before and after treatment. (**e**) The qRT-PCR profiles of *TaVQ25*-*A* in mock (WT and VC) and knockdown lines (1–5). (**f**,**g**) The expressions of the wheat senescence up-regulated genes *TaSAG3* (**f**) and *TaSAG12* (**g**) in *TaVQ25*-*A*-silenced wheat plants. Data and error bars represent means ± SE. Student’s *t*-test, * *p* < 0.05, ** *p* < 0.01, *** *p* < 0.001, and *n* ≥ 20. All of the experiments have been repeated at least three times independently.

To determine the possible role of *TaVQ25*-*A* in regulating leaf senescence, we silenced *TaVQ25*-*A* in wheat using the barley stripe mosaic virus (BSMV)-VIGS method. The severe chlorophyll bleaching phenotype induced by silencing the *TaPDS* gene indicated that the BSMV-VIGS method used in this study was reliable (Figure 4a). A 330-bp fragment of the *TaVQ25*-*A*-specific sequence was amplified to generate the BSMV target site. Because of the high similarity between *TaVQ25*-*A*, *TaVQ25*-*B*, and *TaVQ25*-*D*, it is difficult to clone a unique target sequence only from the *TaVQ25*-*A* cDNA. The two-leaf stage WT wheat plants that were infected by BSMV containing *TaVQ25*-*A*-*A_330_* (pCaBS-α, pCaBS-β, and pCaBS-γbTaVQ25-A-A_330_) or an empty vector (pCaBS-γbLIC). In subsequent analysis, we selected all wheat plants with a decreased *TaVQ25*-*A* transcription level among the infected plants as materials. We observed significantly delayed leaf senescence in *TaVQ25*-*A*-silenced plants but not in VC and WT after 2 d dark treatment (Figure 4b). Senescence-triggered chlorophyll degradation and ion leakage rates in VC and WT were more severe than those in *TaVQ25*-*A*-silenced plants (Figure 4c,d). The expression levels of *TaSAG3* and *TaSAG12* were decreased in the *TaVQ25*-*A*-silenced wheat plants, respectively (Figure 4e,f). These data suggest that *TaVQ25*-*A* is involved in the regulation of senescence in wheat leaves.

**Figure 5:**
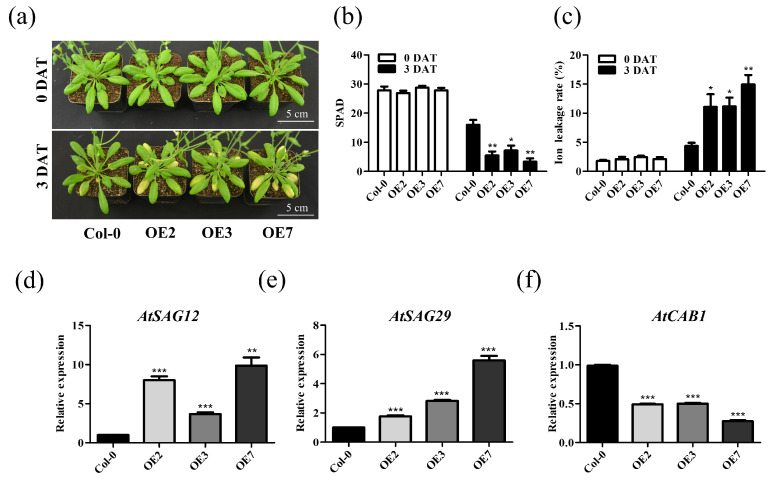
Overexpression of *TaVQ25*-*A* promotes dark-induced leaf senescence. (**a**) Phenotype of 4-week-old Col-0 and *TaVQ25*-*A*-OE plants were treated with darkness for 3 days. (**b**,**c**) Chlorophyll content (**b**) and ion leakage rate (**c**) of leaves from 4-week-old transgenic plants as well as Col-0 before and after dark treatment. (**d**–**f**) Relative expression levels of senescence marker genes in (**a**). Data and error bars represent means ± SE. Student’s *t*-test, * *p* < 0.05, ** *p* < 0.01, *** *p* < 0.001, and *n* ≥ 20. All of the experiments have been repeated at least three times independently.

We compared the 4-week-old *TaVQ25*-*A*-OE and Col-0 plants under dark-induced conditions for 3 d to further analyze the role of *TaVQ25*-*A* in leaf senescence. The *TaVQ25*-*A*-OE leaves showed an early senescence phenotype compared to the Col-0 plants (Figure 5a). Additionally, the chlorophyll content and ion leakage rate were fairly consistent with the phenotypic changes (Figure 5b,c). Furthermore, the expression levels of SAGs (*SAG12* and *SAG29*) and SDG (*CAB1*) were in accordance with the phenotypic alterations between *TaVQ25*-*A*-OE and Col-0 plants, respectively (Figure 5d–f). To confirm these data, detached leaves from 4-week-old *TaVQ25*-*A*-OE and Col-0 plants were incubated with water in the dark for 3 d (Figure S4a). The chlorophyll content and ion leakage were more dramatically altered than in Col-0 (Figure S4b,c). These data collectively demonstrate that *TaVQ25*-*A* positively regulates dark-induced leaf senescence.

### 2.5. TaVQ25-A Is Involved in ABA-Mediated Leaf Senescence

**Figure 6:**
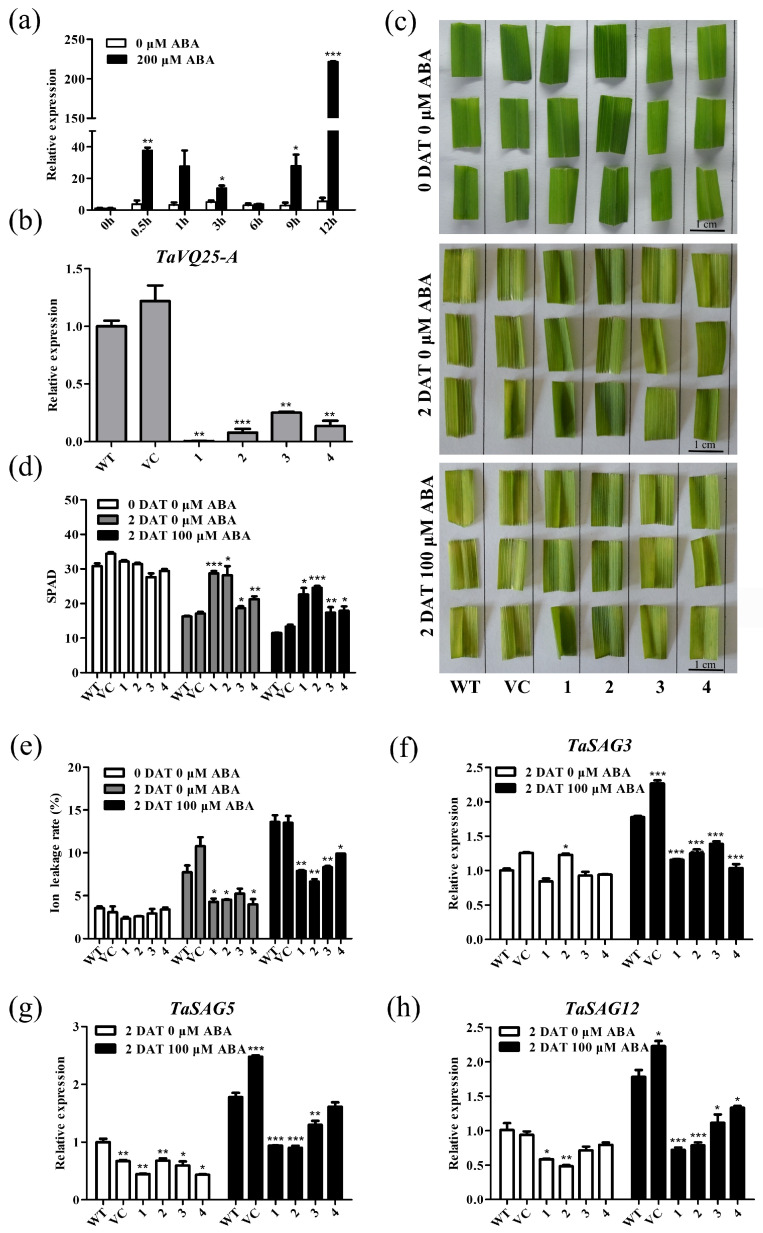
*TaVQ25*-*A*-silenced wheat plants retard the progression of ABA-induced leaf senescence. (**a**) The qRT-PCR analysis of *TaVQ25*-*A* expression in the leaves of wheat seedlings treated with 200 μM ABA at different time points. (**b**) The qRT-PCR profiles of *TaVQ25*-*A* in mock and knockdown lines. (**c**) Phenotypic analysis on the detached leaves of *TaVQ25*-*A*-silenced and control plants under 100 μM ABA treatment. (**d**,**e**) Chlorophyll content (**d**) and ion leakage rate (**e**) of *TaVQ25*-*A*-silenced and control plants before and after ABA treatment. (**f**–**h**) Relative expression levels of the senescence marker genes *TaSAG3* (**f**), *TaSAG5* (**g**), and *TaSAG12* (**h**) in leaves treated with ABA. Data and error bars represent means ± SE. Student’s *t*-test, * *p* < 0.05, ** *p* < 0.01, *** *p* < 0.001, and *n* ≥ 20. All of the experiments have been repeated at least three times independently.

To uncover the underlying mechanism of *TaVQ25*-*A*-related leaf senescence, we searched the 2 kb promoter region of *TaVQ25*-*A* to identify the *cis*-elements on PlantCARE1 (http://bioinformatics.psb.ugent.be/webtools/plantcare/html/) (accessed on 10 November 2021) (Figure S5). Notably, five ABA-responsive elements (ABRE) were found in the *TaVQ25*-*A* promoter region (−928, −853, −175, −163, and −90 bp). Therefore, we hypothesized that TaVQ25-A regulates leaf senescence via the ABA pathway. We examined the expression pattern of *TaVQ25*-*A* after treatment with 200 µM ABA. The transcription levels of *TaVQ25*-*A* were at the maximum level after 12 h of treatment (Figure 6a). We then analyzed the ABA-induced leaf senescence among the *TaVQ25*-*A*-silenced wheat plants and the controls (Figure 6b). ABA-induced leaf senescence was assessed via the detached leaves of the *TaVQ25*-*A*-silenced wheat plants and the controls. After treatment, leaf senescence triggered by ABA appeared earlier in the controls than in the *TaVQ25*-*A*-silenced wheat plants (Figure 6c). Concomitantly, higher chlorophyll content and lower ion leakage rates were detected in the ABA-treated *TaVQ25*-*A*-silenced plants than in the controls (Figure 6d,e). Furthermore, the expression of *TaSAG3*, *TaSAG5*, and *TaSAG12* was reduced in the *TaVQ25*-*A*-silenced plants (Figure 6f–h).

**Figure 7:**
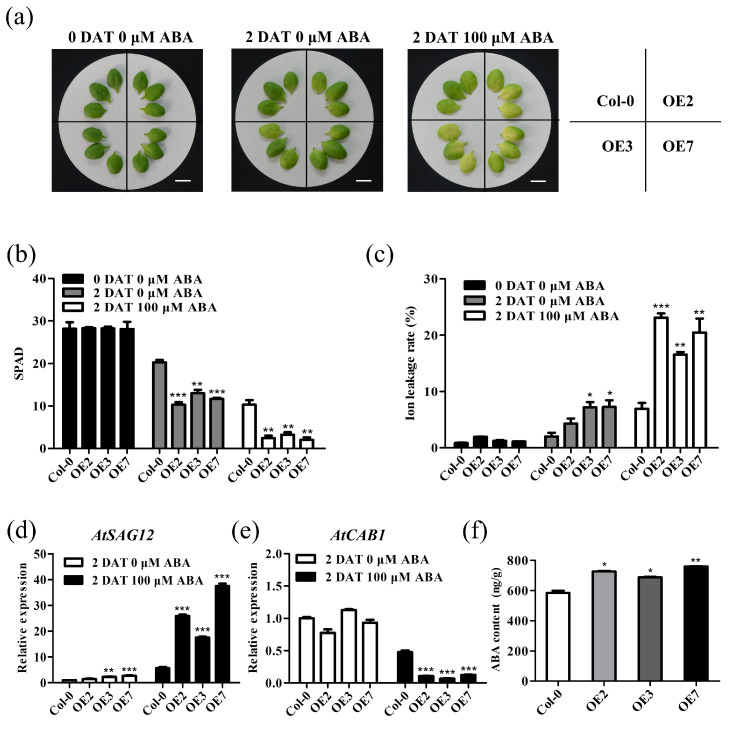
Overexpression of *TaVQ25*-*A* accelerates ABA-induced leaf senescence. (**a**) Phenotype of 3-week-old Col-0 and *TaVQ25*-*A*-OE leaves treated with or without 100 μM ABA for 2 days. (Scale bar = 1 cm). (**b**,**c**) Measurement of chlorophyll content (**b**) and ion leakage rate (**c**) of the detached leaves in (**a**). (**d**,**e**) Expression levels of senescence-response genes, including *AtSAG12* (**d**) and *AtCAB1* (**e**), in leaves treated with ABA or without ABA for 2 days. (**f**) Endogenous ABA content was measured in TaVQ25-A-OEs plants and Col-0 enzyme-linked immunosorbent assay. Data and error bars represent means ± SE. Student’s *t*-test, * *p* < 0.05, ** *p* < 0.01, *** *p* < 0.001, and *n* ≥ 20. All of the experiments have been repeated at least three times independently.

In addition, we treated the non-senescent fifth or sixth rosette leaves of 4-week-old *TaVQ25*-*A*-OE and Col-0 plants with 100 μM ABA for 2 d. ABA-induced leaf senescence appeared in both *TaVQ25*-*A*-OE and Col-0 plants (Figure 7a), but lower chlorophyll content and more severe ion leakage rates were found only in the *TaVQ25*-*A*-OE plants (Figure 7b,c). The transcription data for *AtSAG12* and *AtCAB1* coincided with the phenotypic changes between *TaVQ25*-*A*-OE and Col-0 plants (Figure 7d,e). To further clarify the effects of TaVQ25-A on ABA biosynthesis, we quantified the ABA content in the leaves of *TaVQ25*-*A*-OE plants and Col-0. Using enzyme-linked immunosorbent assay, we found that ABA content was elevated in the leaves of *TaVQ25*-*A*-OE lines when compared with that in Col-0. These findings strongly support our hypothesis that *TaVQ25*-*A* is involved in the regulation of leaf senescence via the ABA pathway.

### 2.6. TaVQ25-A Interacts with TaWRKY133 In Vitro and In Vivo

**Figure 8:**
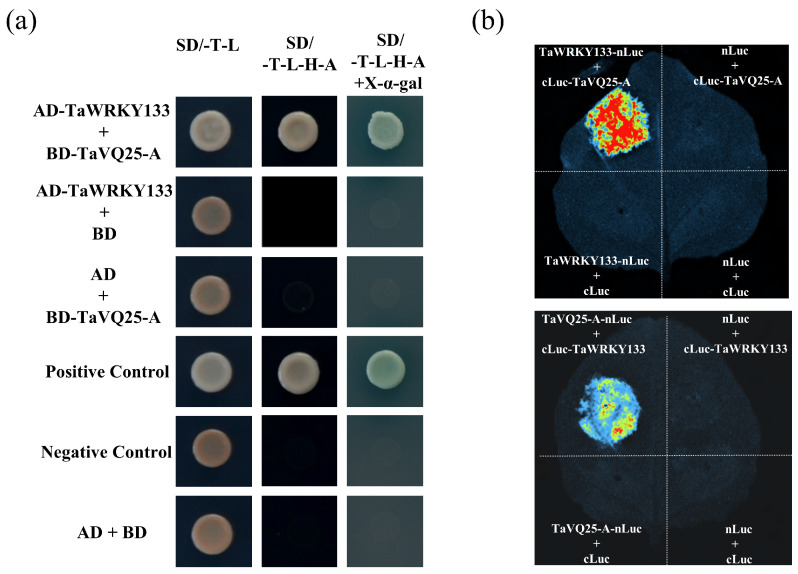
Interaction of TaVQ25-A with TaWRKY133. (**a**) Interaction of TaVQ25-A with TaWRKY133 in yeast. The constructs pGADT7-TaWRKY133 and pGBKT7-TaVQ25-A were co-transformed in yeast strain AH109, then performed on SD/-T-L (SD/-Trp-Leu) and SD/-T-L-H-A (SD/-Trp/-Leu/-His/-Ade) plates. Positive control: pGADT7-T + pGBKT7-53; negative control: pGADT7-T + pGBKT7-Lam and AD + BD. *n* ≥ 20. (**b**) TaVQ25-A interacted with TaWRKY133 in the firefly luciferase complementation imaging (LCI) assay. *n* ≥ 20.

In *Arabidopsis*, the majority of AtVQ proteins interact with the WRKY transcription factors. To further explore the mechanism of the TaVQ25-A-regulated leaf senescence, we screened a WRKY transcription factor called TaWRKY133 through a yeast screening library. Previously, TaWRKY133 has been reported to negatively regulate plant drought resistance by repressing the expression of some ABA-related genes, including ABF1, ABA2, and ABI1, which led us to test the interaction between TaVQ25-A and TaWRKY133 [38]. The yeast cells harboring the recombinant plasmids (pGADT7-TaWRKY133 + pGBKT7-TaVQ25-A, pGADT7-TaWRKY133 + pGBKT7, pGADT7 + pGBKT7-TaVQ25-A, pGADT7-T + pGBKT7-T53, and pGADT7-T + pGBKT7-lam) grew normally on the SD/-Trp/-Leu medium (Figure 8a). However, on the SD/-Trp/-Leu/-His/-Ade medium, the yeast cells of the positive control and experimental groups grew normally and turned blue in the presence of X-α-gal, whereas those of the negative control group did not grow normally. In addition, we certified this interaction using a firefly luciferase complementation imaging (LCI) assay in tobacco leaves. Fluorescent signals were only captured in tobacco leaves that harbored the combinations of TaWRKY133-nLuc + cLuc-TaVQ25-A or TaVQ25-A-nLuc + cLuc-TaWRKY133, and those of the negative control group had no fluorescent signal (Figure 8b). Thus, TaVQ25-A interacted with the TaWRKY133 transcription factor. These results demonstrate that TaVQ25-A and TaWRKY133 interact with each other in vitro and in vivo.

## 3. Discussion

Leaf senescence is a complex and highly programmed process, and it is controlled by various regulatory networks [39]. The initiation of senescence is the result of continuous integration between internal changes and environmental signals at the cellular, tissue, and organ levels [40]. The senescence of leaves is related to the development and maturity of fruits and seeds and ends with the complete degradation of organelles and macromolecules [41]. Leaf photosynthesis is essential to maximizing carbohydrate levels in seeds or fruits, so delaying senescence contributes to increased yields. In addition, appropriate and effective senescence regulation is conducive to facilitating the source-to-sink allocation [42].

VQ proteins have been identified in many plant species, and their functional roles in some biological events have been identified. As both positive and negative regulators, VQ proteins regulate multiple plant growth and development processes, such as endosperm growth, seed size, and responses to biotic and abiotic stresses, through interacting with different transcription factors [43,44,45]. However, the functional role of the VQ family members in leaf senescence regulation remains obscure.

Here, we identified new candidate genes that regulate leaf senescence in wheat by searching our wheat transcriptome database. We demonstrated that the expression of *TaVQ25*-*A* was higher in senescent flag leaves than in nonsenescent flag leaves and that a high expression of *TaVQ25*-*A* was also detected at the tip of a single senescent leaf. Moreover, we showed that the overexpression of *TaVQ25*-*A* promoted leaf senescence under natural and dark treatment conditions in *Arabidopsis*, and *TaVQ25*-*A*-silenced wheat showed a significantly delayed leaf senescence phenotype (Figure 4 and Figure 5). These data prove that *TaVQ25*-*A* acts as a senescence-associated gene and an activator of leaf senescence. To date, few senescence-related VQs have been mechanistically studied. VQ proteins SIB1 and SIB2 participate in ABA-mediated leaf senescence and seed germination by inhibiting WRKY75 in *Arabidopsis* [32]. Overexpression of *ZmVQ52* accelerated leaf senescence in *Arabidopsis* [23]. MdVQ10 accelerates wound-induced leaf senescence by cooperating with MdWRKY75, which is also affected by MdCML15 and MdJAZs in apples [46]. In wheat, there are 113 putative VQs in total; however, little is known about how these VQs impact leaf senescence [37]. In this study, we identified a VQ gene *TaVQ25-A* and revealed the intrinsic relationship between TaVQ25-A, TaWRKY133, and ABA pathway in the regulation of leaf senescence, providing a new candidate gene for modulating the leaf senescence process and molecular breeding in wheat.

Many transcription factors have been reported to regulate leaf senescence via hormone pathways [47,48,49]. As a sesquiterpenoid hormone, ABA regulates various processes, such as seed dormancy and germination, root and stem growth, leaf senescence, stomatal closure, fruit ripening, and responses to biotic and abiotic stress [50]. The endogenous ABA content and the ABA signaling pathway components play important roles in the regulation of leaf senescence [51]. Many ABA-related TFs, including AtNAP, OsNAC2, SlNAP2, and GhNAP, play vital roles in the regulation of leaf senescence [11,52,53,54]. Recently, TaNAC69-B promotes leaf senescence by affecting ABA levels, suggesting that ABA-associated fine-tuning at the transcriptional level is important for the modulation of leaf senescence in wheat [55]. Notably, we detected higher ABA levels in *TaVQ25*-*A*-OE *Arabidopsis* lines than that in Col-0. Under ABA treatment, we observed that leaf senescence in *TaVQ25*-*A*-silenced wheat plants was relatively insensitive to ABA. Meanwhile, *TaVQ25*-*A*-OE *Arabidopsis* plants showed more severe ABA-induced leaf senescence than Col-0. Thus, our results indicated that *TaVQ25*-*A* positively regulates leaf senescence by interacting with the ABA pathway. More importantly, the positive role of TaVQ25-A in promoting leaf senescence in Arabidopsis and wheat implies that the underlying mechanism of TaVQ25-A-regulated leaf senescence is partially conserved in monocots and dicots.

Generally, VQs cooperate with transcription factors to regulate the transcription of downstream genes [21]. Our study showed that TaVQ25-A was concentrated in the nuclei and cytoplasm, indicating that TaVQ25-A possibly participates in the transcription events. Intriguingly, we showed that TaVQ25-A possessed transcriptional repression activity, which led us to investigate the delicate relationship between TaVQ25-A and the ABA pathway in leaf senescence. Through Y2H screening and LCI assay, we identified TaWRKY133 as a protein–protein interaction partner of TaVQ25-A. TaWRKY133 negatively regulates plant drought resistance and represses some ABA-related genes [38]. Although there is no evidence of TaWRKY133 impacting leaf senescence under natural conditions, TaWRKY133 shows great potential in the regulation of stress-induced senescence. Whether this interaction of TaVQ25-A with TaWRKY133 could attenuate the suppression of TaWRKY133 on ABA-related genes during the senescence process needs to be further investigated. Moreover, mutants of *tavq25-a* and *tawrky133* generated by the CRISPR-Cas9-mediated gene-editing approach will be necessary to evaluate the mechanistic details of TaVQ25-A-TaWRKY133 working module more precisely in wheat leaf senescence. It is widely acknowledged that ABA content is accumulated during the senescence process and that excessive ABA levels promote leaf senescence onset [56]. Our data showed that the overexpression of *TaVQ25-A* elevated ABA content in *Arabidopsis* and that the expression of *TaVQ25* is inducible under ABA treatment, suggesting that there is a positive feedback loop between TaVQ25-A, ABA pathway, and leaf senescence. How TaVQ25-A affects ABA biosynthesis in wheat is still uncertain and needs to be analyze in future. In addition, analysis of *TaVQ25*-*A* haplotypes will be very helpful when it comes to using this gene in molecular breeding.

Briefly, we identified a VQ family member, TaVQ25-A, as a positive regulator of leaf senescence progression. TaVQ25-A promotes leaf senescence predominantly by interacting with the ABA pathway and TaWRKY133 and further impacts ABA biosynthesis. Our data provide a new candidate target for molecular breeding through the optimization of the senescence process in wheat.

## 4. Materials and Methods

### 4.1. Plant Materials and Growth Conditions

The hexaploid wheat cultivar ‘cv. Chinese Spring’ was used for analyzing the expression profile of *TaVQ25*-*A*. Flag leaves were collected at different developmental stages from various tissues in the field. Wheat plants were grown in a greenhouse at 25 °C under long day conditions (16 h light/8 h dark cycle) and 60% relative humidity for BSMV-mediated gene silencing. The 7-day-old etiolated seedlings of ‘Jinhe 311’ were used to generate wheat protoplasts. The *Arabidopsis* ecotype Col-0 was obtained from *Arabidopsis* Biological Resource Center (ABRC, https://abrc.osu.edu) (accessed on 25 June 2019). *Arabidopsis* plants were grown in a greenhouse at 22 °C under long day conditions (16 h light/8 h dark cycle) with 60% relative humidity. Col-0 was used for the transgenic analysis of *TaVQ25*-*A*. The primers used for the identification of clones are listed in Table S1.

### 4.2. Plasmid Construction and Plant Transformation

A full-length CDS of *TaVQ25*-*A* was constructed in the pCAMBIA1300-Flag vector driven by the 35S promoter. The above vector was transformed into Col-0 by the *Agrobacterium tumefaciens* strain GV3101 using the floral dip transformation method [57]. TaVQ25-A-Flag fusions in transgenic lines were detected via a Western blot assay with a Flag antibody. The full-length CDS of *TaVQ25*-*A* was sub-cloned into pSAT-GAL4BD, pGBKT7, and PUC19 vectors to test transcriptional activity and subcellular localization. A 330 bp fragment of *TaVQ25*-*A* was constructed in the pCaBS-γbLIC vector to generate *TaVQ25*-*A* BSMV-VIGS constructs.

### 4.3. Chlorophyll Content and Ion Leakage Assay

The chlorophyll content was detected using a SPAD502 Plus Chlorophyll Meter (Minolta Corporation, Tokyo, Japan). Leaves were collected, placed in 5 mL of deionized water, and vacuumed for 0.5 h. The conductivity was measured before and after boiling for 15 min. After subtracting the conductivity of the water, the ion leakage rate was calculated using the ratio of the conductivity of leaves before and after being boiled in deionized water.

### 4.4. qRT-PCR Analysis

Total RNA was extracted from *Arabidopsis* and wheat using Trizol reagent (TaKaRa, 9109, Tokyo, Japan). Total RNA (500 ng) was used to generate cDNA using 5× HiScriptII qRT SuperMixII (R223-01, Vazyme Biotech Co., Ltd., Nanjing, Jiangsu, China). Real-time PCR was performed using SYBR qPCR master mix (Q711-02, Vazyme Biotech Co., Ltd., Nanjing, Jiangsu, China) with the CFX96TM Real-Time system (Bio-Rad, Hercules, CA, USA). All primers used in this study are listed in Table S1. The expression levels of the target genes were normalized to the expression levels of the internal controls, i.e., *TaACTIN* in wheat and *AtUBC30* in *Arabidopsis*.

### 4.5. Subcellular Localization Assay

The CDS of *TaVQ25*-*A* was sub-cloned into the PUC19 vector to generate the 35S:TaVQ25-A-GFP construct. The 35S:GFP empty vector was used as a control. The CDS of *TaWRKY42*-*B* [58], a nuclear localization gene, was sub-cloned into the pYJmCherry vector as a nuclear localization control. GFP and mCherry signals were observed under a laser confocal microscope (Olympus, FV3000, Tokyo, Japan) 12 h after transient expression of these plasmids in wheat protoplasts.

### 4.6. Transcriptional Activation Assay

The CDS of *TaVQ25*-*A* was sub-cloned into the pGBKT7 vector. The plasmid was transformed into the AH109 yeast strain, and the empty pGBKT7 vector was used as a negative control. The yeast cells were first cultured on a selective medium (SD) without tryptophan (SD/-T), and the obtained positive yeast cells were then grown on SD plates without tryptophan, histidine, or adenine (SD/-T-L-H) and SD/-T-L-H plates containing X-α-D-galactosidase (X-α-gal) to observe their transcriptional activation activity.

The CDS of *TaVQ25*-*A* was sub-cloned into the pSAT-GAL4BD vector and transformed into wheat protoplasts. The transcription activation activity of *TaVQ25*-*A* was represented by the ratio of firefly luciferase (Luc), the reporter, to *Renilla* luciferase (Ren), the internal reference gene, and was measured using a microplate cold light detector (LB960, Berthold, Wildbad, Germany).

### 4.7. Virus-Induced Gene Silencing (VIGS) Assay

A ligation-independent cloning (LIC) strategy was used for the BSMV α, β, or γ (or derivatives), which were generously provided by Prof. Dawei Li of China Agricultural University, Beijing, China. The 330-bp gene fragment was constructed on the pCaBS-γ vector. The BSMV α, β, and γ and recombinant plasmids were transformed into the A. tumefaciens strain GV3101 to be used for infiltrating tobacco leaves for mechanical inoculation onto two-leaf stage wheat seedlings [59,60].

### 4.8. Yeast Two-Hybrid (Y2H) Assays

The CDS of *TaVQ25*-*A* was added to pGBKT7 to generate BD-TaVQ25-A. The CDS of *TaWRKY133* was added to the pGADT7 vector to generate AD-TaWRKY133. The recombinant vectors were transformed into the AH109 yeast strain. Empty plasmids were used as controls. The growth of transformed yeast cells on SD/-T-L (SD/-Trp-Leu) and SD/T-L-H-A (SD/-Trp/-Leu/-His/-Ade) media was used to reflect the strength of the protein–protein interaction.

### 4.9. ABA Treatment and Quantification of ABA Content

Detached leaves of *TaVQ25*-*A*-silenced and control plants and the fifth and sixth rosette leaves of 4-week-old Arabidopsis plants were placed on filter paper and incubated with 100 μM ABA solution for 2 days. The treated leaves were collected and stored at 80 °C for future physiological analysis and RNA extraction. For endogenous ABA content analysis, leaves of 4-week-old *Arabidopsis* plants were used to measure the endogenous ABA content. ABA was extracted from 500 mg frozen leaf powder according to the method of plant ABA enzyme-linked immunosorbent assay (ELISA) kit (YM-0052, Shanghai Yuanmu Biotechnology Co., Ltd., Shanghai, China). The resulting supernatant ABA concentration was determined by a high-performance liquid chromatograph using Multiskan™ GO Microplate Photometer (1,410,101, Thermo Fisher, Shanghai, China).

### 4.10. Luciferase Complementation Imaging (LCI) Assay

The LCI assay was performed as previously described [61]. Both TaVQ25-A and TaWRKY133 were fused to the C-terminal half of luciferase (cluc) and the N-terminal half of LUC (nluc), respectively. *Nicotiana benthamiana* leaves were co-infiltrated with the vectors and incubated in the dark for 24 h at 25 °C. Leaves were sprayed with the luciferase substrate and incubated at ambient temperature in the dark for 5 min. Images were captured using a low-light, cooled CCD imaging device.

### 4.11. Statistical Analysis

The error bars represent the standard error (SE). Analysis of the significance level was performed according to Student’s *t*-test method at * *p* < 0.05, ** *p* < 0.01, and *** *p* < 0.001 using SPSS Statistics 20.0 software. The figures were generated using GraphPad Prism 7 software.

## Data Availability

The data presented in this study are available in the article or Supplementary Materials.

## Supplementary Materials

The following supporting information can be downloaded at: https://www.mdpi.com/article/10.3390/ijms241813839/s1.

## Accession Numbers

Sequence data produced in this study can be accessed via EnsemblPlant (https://plants.ensembl.org/Triticum_aestivum/Info/Index) (accessed on 15 November 2020), WheatOmics (http://202.194.139.32/) (accessed on 15 November 2020) and Tair (https://www.arabidopsis.org/) (accessed on 5 March 2021) websites under the following accession numbers: *TaACTIN* (TraesCS1A02G274400), *TaSAG3* (TraesCS5A02G120000), *TaSAG5* (TraesCS5D02G065100), *TaSAG12* (TraesCS5B02G368300), *TaVQ25*-*A* (TraesCS4A02G290800), *TaVQ25*-*B* (TraesCS4B02G023200) and *TaVQ25*-*D* (TraesCS4D02G021000), *AtUBC30* (AT5G56150), *AtSAG12* (AT5G45890), *AtSAG29* (AT5G13170), *AtCAB1* (AT1G29930).
