# Supplementary material for: Wheat VQ Motif-Containing Protein VQ25-A Facilitates Leaf Senescence via the Abscisic Acid Pathway
Source: Int J Mol Sci. 2023 Sep 8;24(18):13839. doi: 10.3390/ijms241813839 (PMC10531066; doi:10.3390/ijms241813839)
Supplement: Supplementary file 1 [file ijms-24-13839-s001.zip › Supplementary Figures TaVQ25-Meng et.al.pdf]

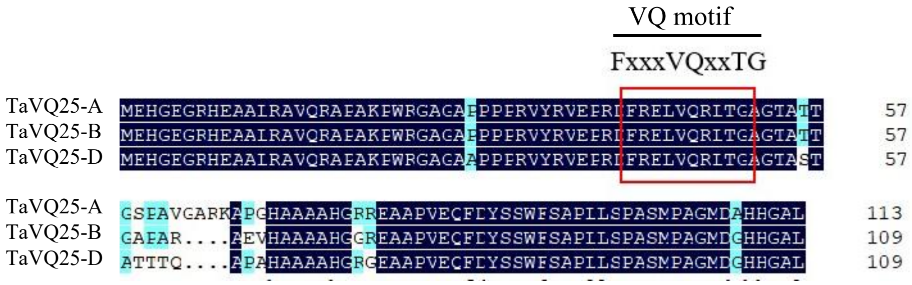

Figure S1. Alignment of TaVQ25-A, TaVQ25-B and TaVQ25-D amino acid sequences**.**

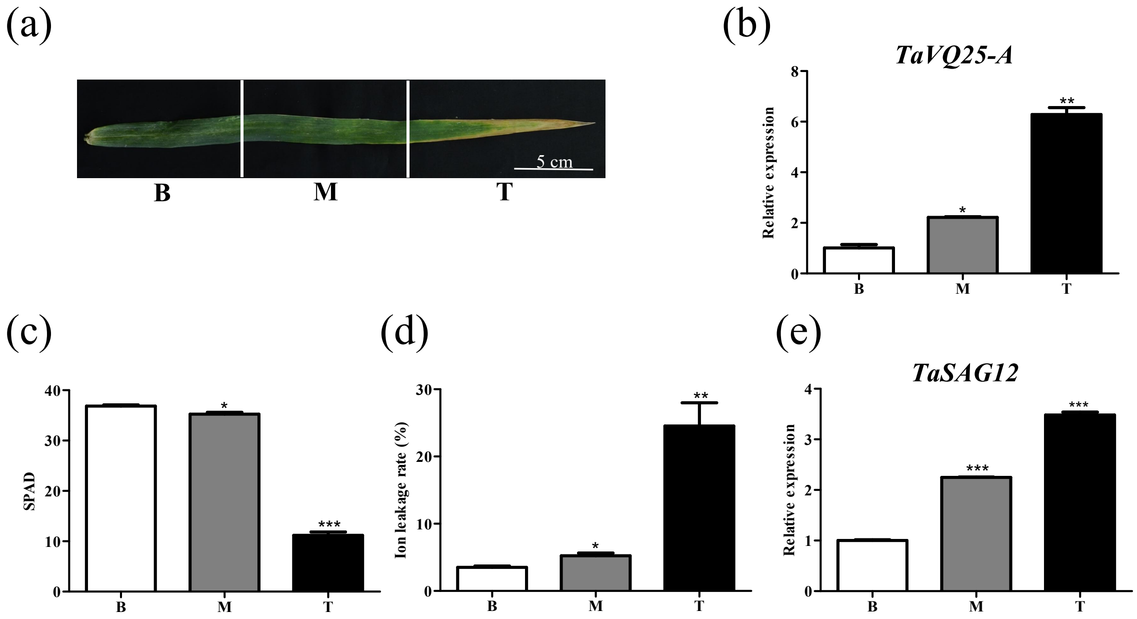

Figure S2. *TaVQ25-A* expression in different parts of a senescent wheat flag leaf.
(a) Different parts (B: base, M: middle, and T: tip) of a senescent wheat leaf.
(b) Expression of *TaVQ25-A* in different parts of a senescent wheat leaf.
(c, d) Chlorophyll content (c) and ion leakage rate (d) of different parts of a senescent wheat leaf.
(e) Expression of senescence up-regulated marker gene *TaSAG12* in different parts of a senescent wheat leaf. Data and error bars represent means ±SE. Student's *t*-test, $*P < 0.05$, $**P < 0.01$, $***P < 0.001$. All of the experiments have been repeated at least three times independently.

(a) (b)

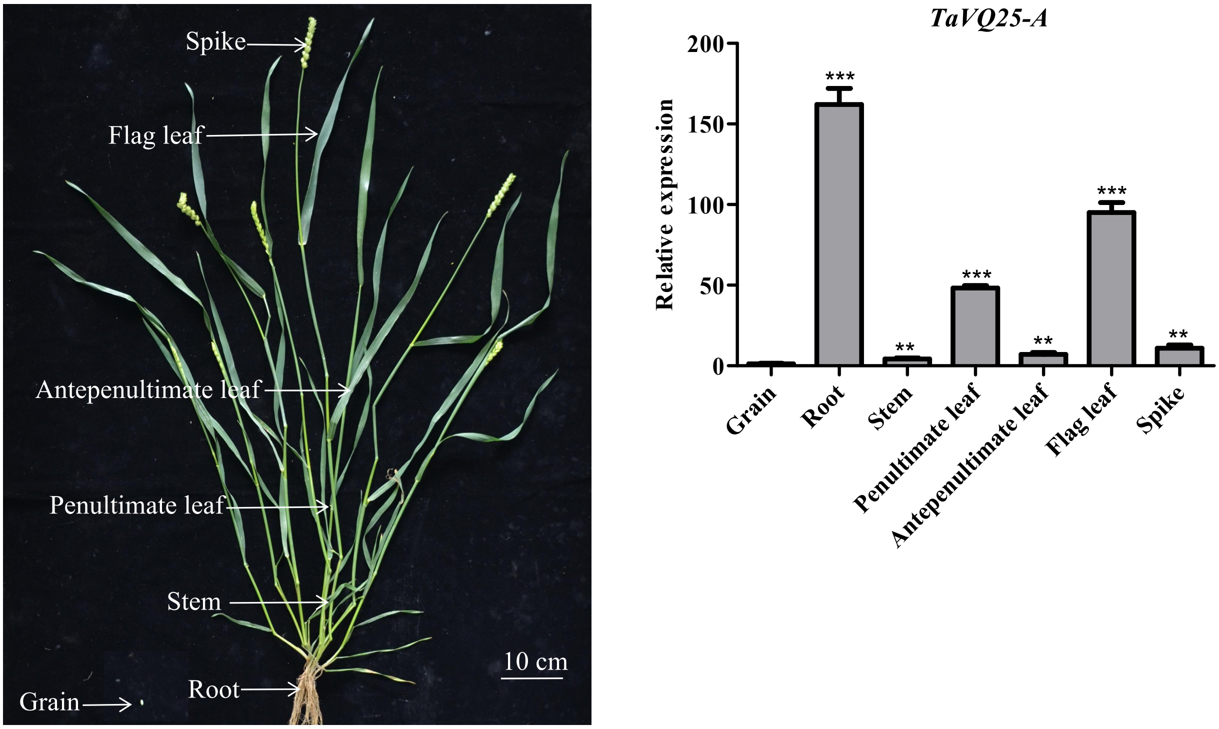

Figure S3. Expression pattern of *TaVQ25-A*.
(a) Different tissues of a wheat plant.
(b) Expression of *TaVQ25-A* in different wheat tissues.
Data and error bars represent means ±SE. Student's *t*-test, *P < 0.05, **P < 0.01, ***P < 0.001. All of the experiments have been repeated at least three times independently.

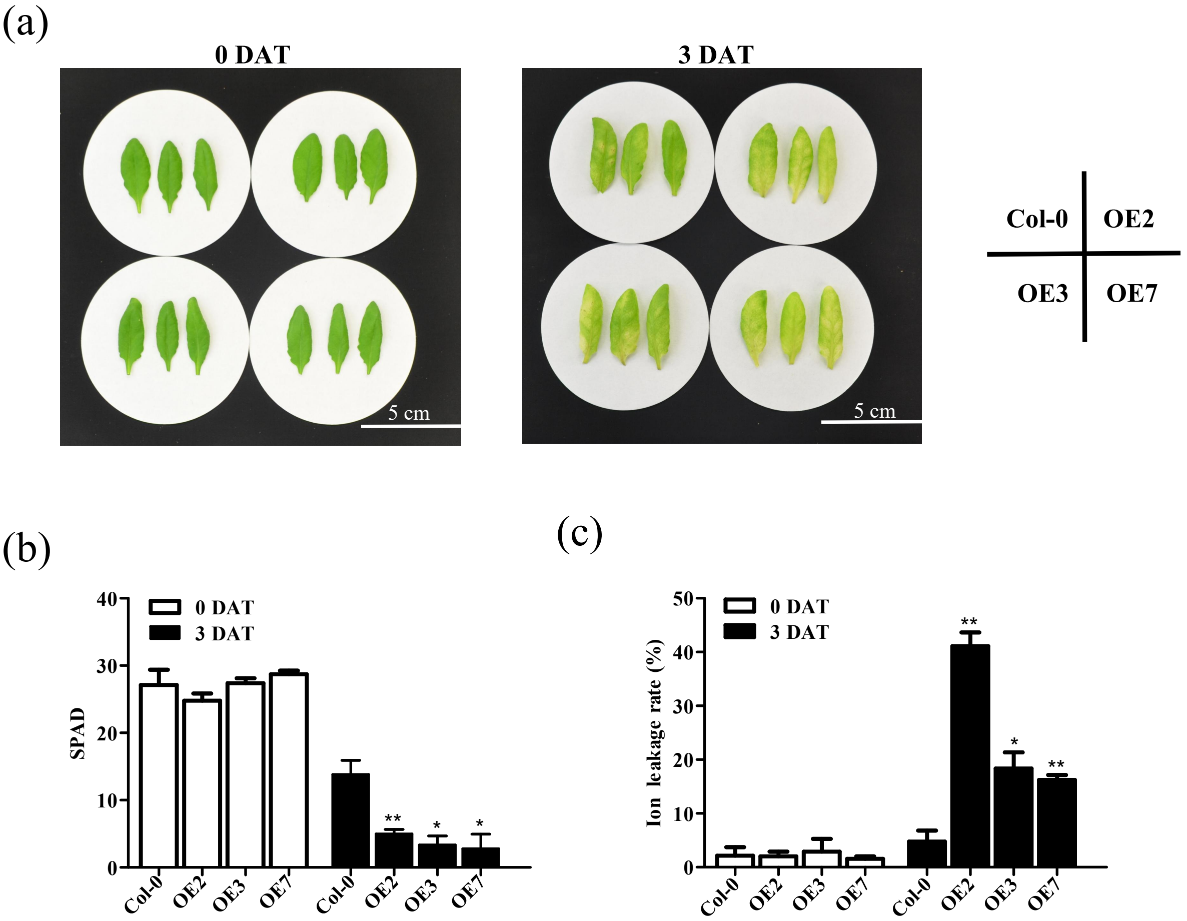

Figure S4. Overexpression of *TaVQ25-A* promotes dark-induced leaf senescence.
(a) Detached leaves of 4-week-old Col-0 and *TaVQ25-A*-OE plants were treated with darkness for 3 days.
(b, c) Chlorophyll content (b) and ion leakage rate (c) of leaves from 4-week-old transgenic plants as well as Col-0 before and after dark treatment.
Data and error bars represent means ±SE. Student's *t*-test, *$P < 0.05$, **$P < 0.01$, ***P < 0.001. All of the experiments have been repeated at least three times independently.

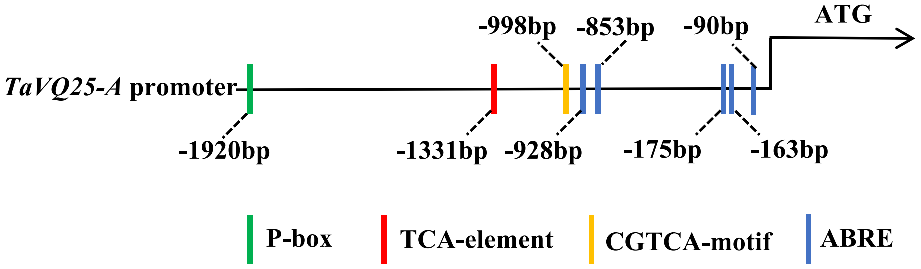

Figure S5. Promoter analysis of *TaVQ25-A* in PlantCARE.
The localization of *cis*-elements in *TaVQ25-A* promoter region. P-box is involved in GA responsiveness, TCA-element is involved in SA responsiveness, CGTCA-motif is involved in MeJA responsiveness, and ABRE is involved in ABA responsiveness.
