# Supplementary material for: Wheat VQ Motif-Containing Protein VQ25-A Facilitates Leaf Senescence via the Abscisic Acid Pathway
Source: Int J Mol Sci. 2023 Sep 8;24(18):13839. doi: 10.3390/ijms241813839 (PMC10531066; doi:10.3390/ijms241813839)
Supplement: Supplementary file 1 [file ijms-24-13839-s001.zip › Table S1 TaVQ25-Meng et.al.pdf]

Table S1. Primers used in this study

Primers	Sequence (5' to 3')
TaSAG3 (TraesCS5A02G120000)-F for qRT-PCR	CGACATCCGACGATTCAAC
TaSAG3-R	TCGCACCACCATCCATTC
TaSAG5 (TraesCS5D02G065100)-F for qRT-PCR	CGGCTGCGAAGTTGGTTAC
TaSAG5-R	ACCTGCTCCTGAGATAATGG
TaSAG12(TraesCS5B02G368300)-F for qRT-PCR	GCCAACTTCCAGCACTACAGG
TaSAG12-R	AGAACAATGAACCAAGCACACC
TaVQ25-A (TraesCS4A02G290800)-F for qRT-PCR	CGTGGGAGCGAGGAAGG
TaVQ25-A-R	CGAGCGGCGACCGTAG
TaACTIN (TraesCS1A02G274400)-F for qRT-PCR	TGCTATCCTTCGTTTGGACCTT
TaACTIN-R	AGCGGTTGTTGTGAGGGAGT
AtSAG12 (AT5G45890)-F for qRT-PCR	GGATGTCCCGGTTAATGATG
AtSAG12-R	TGGAAATCAAAACCACCTCC
AtSAG29 (AT5G13170)-F for qRT-PCR	GCCACCAGGGAGAAAAGG
AtSAG29-R	CCACGAAATGTGTTACCATTAGAA
AtCAB1(AT1G29930)-F for qRT-PCR	TGTGCTGCACTACTCAACCT
AtCAB1-R	TCCAAGGACTTCAGATGCCG
AtUBC30 (AT5G56150)-F for qRT-PCR	CAAATCCAAAACCCTAGAAACCGAA
AtUBC30-R	ATCTCCCGTAGGACCTGCACTG
TaVQ25-A-F for CDs	ATGGAGCACGGCGAGGGGA
TaVQ25-A-R	CTACAGCAGAGCGCCATGGT
TaVQ25-A-F for BSMV-VIGS	AAGGAAGTTTAAATGGAGCACGGCGAGGGGAG
TaVQ25-A-R	AACCACCACCACCGTCTACAGCAGAGCGCCATGGTG
TaWRKY133 (TraesCS3B02G379200)-F for CDs	ATGACAACCTCGTCGTCAGGGA
TaWRKY133-R	TCAGTACTGGGAGTTCTGGAAGA
